# Transcriptome and weighted gene co-expression network analysis identify hub genes and pathways in rat kidneys after deep hypothermic circulatory arrest

**DOI:** 10.1080/0886022X.2026.2635286

**Published:** 2026-03-10

**Authors:** Lei Wang, Ziyan Lin, Yuzuo Lin, Qingtong Wu, Guodong Zhong, Liangwan Chen

**Affiliations:** ^a^ Department of Cardiovascular Surgery, Fujian Medical University Union Hospital, Fuzhou, China; ^b^ Union College of Clinical Medicine, Fujian Medical University, Fuzhou, China; ^c^ Department of Pathology, Fujian Province Second People’s Hospital: The Second Affiliated Hospital of Fujian University of Traditional Chinese Medicine, Fuzhou, China; ^d^ Engineering Research Center of Tissue and Organ Regeneration, Fujian Province University, Fuzhou, China

**Keywords:** Transcriptome profiling, weighted gene co-expression network analysis, hub genes, deep hypothermic circulatory arrest, acute kidney injury

## Abstract

**Background:**

Acute kidney injury (AKI) is a frequent and severe complication following aortic dissection repair surgery that utilizes deep hypothermic circulatory arrest (DHCA). This study aimed to identify key hub genes and elucidate the molecular pathways involved in DHCA-associated AKI (DHCA-AKI).

**Methods:**

A rat model of DHCA was established, and kidney tissues were harvested for high-throughput transcriptome sequencing. Differentially expressed genes (DEGs) between DHCA and control groups were identified using three independent analytical methods (DESeq2, limma, edgeR). Hub genes were subsequently screened by integrating weighted gene co-expression network analysis (WGCNA) and protein-protein interaction (PPI) network analysis. The iRegulon plugin was used to predict upstream regulators. Kidney function markers and the expression levels of identified hub genes were experimentally validated.

**Results:**

We identified 154 significant DEGs (80 up- and 74 down-regulated) and 209 key module genes *via* WGCNA. Ten hub genes were pinpointed: *Jun*, *Atf3*, *Cebpb*, *Il-1β*, *Nfκbia*, *Ptgs2*, *Cxcl1*, *Nfκbiz*, *Zfp36*, and *Tnfaip3*. These genes are predominantly associated with inflammatory responses and apoptotic processes. RELA and ATF3 were identified as potential upstream transcriptional regulators. Experimental validation confirmed significant kidney impairment and the substantial upregulation of these hub genes at both mRNA and protein levels following DHCA.

**Conclusion:**

DHCA markedly alters the transcriptional landscape in rat kidney tissues. Hub genes and pathways related to inflammation and apoptosis may constitute central pathogenic mechanisms and represent promising therapeutic targets for DHCA-AKI.

## 1. Introduction

Deep hypothermic circulatory arrest (DHCA) is a specialized cardiopulmonary bypass (CPB) technique primarily used in surgeries for aortic dissection. This technique involves halting systemic circulation under deep hypothermia (<20 °C), providing a bloodless surgical field while effectively reducing the overall metabolic rate to protect organ function. The kidneys are among the organs with the highest metabolic rates, second only to the heart [1]. Although organ metabolism is reduced during DHCA, injury related to hypothermia itself, microcirculatory disturbances, renal ischemia-hypoxia, and reperfusion injury remain significant concerns following circulatory arrest and subsequent reperfusion. Studies using rabbit DHCA models have shown an association between this technique and renal dysfunction, characterized by early elevations in serum cystatin C and neutrophil gelatinase-associated lipocalin (NGAL), reduced renal antioxidant capacity, and apoptosis of renal tubular epithelial cells [2]. Furthermore, while antegrade cerebral perfusion techniques during hypothermic circulatory arrest can mitigate the risk of cerebral hypoperfusion, it is important to note that the kidneys remain in a non-perfused state throughout the arrest period. These factors collectively contribute to the occurrence of postoperative acute kidney injury (AKI).

AKI is one of the most prevalent complications following aortic dissection (AD) repair surgery, with an incidence rate of ∼50% [3], which surpasses that observed in other types of cardiac surgeries. The 30-day mortality rate for patients experiencing postoperative AKI stands at 11.8%, and their risk of death is 3.12 times greater than that of patients without AKI [3]. Currently, effective pharmacological prevention or treatment strategies are lacking, and the prognosis for patients requiring renal replacement therapy remains poor [4]. Therefore, elucidating the molecular mechanisms underlying DHCA-associated AKI (DHCA-AKI) is crucial for developing targeted therapies.

Advances in high-throughput sequencing and bioinformatics offer powerful tools for uncovering disease mechanisms. While previous studies have investigated miRNA profiles in DHCA-AKI [5], comprehensive transcriptomic alterations and their associated pathways in kidney tissues post-DHCA remain poorly characterized. Understanding these changes is vital for identifying specific biomarkers and therapeutic targets.

In this study, we established a rat model of DHCA to investigate the transcriptomic response in kidney tissues. This model minimized confounding surgical effects and enabled the clarification of DHCA-AKI mechanisms. Through integrated differential expression analysis, Weighted gene co-expression network analysis (WGCNA), and Protein-protein interaction (PPI) network construction, we aimed to identify key hub genes and pathways in DHCA-AKI, offering insights into its pathogenesis and potential therapeutic strategies.

## 2. Materials and methods

### 2.1. Construction of a rat DHCA model

All animal procedures were approved by the Animal Ethics Committee of Fujian Medical University (IACUC FJMU 2024-Y-1767) and complied with international standards. Twelve 10-week-old specific pathogen-free male Sprague-Dawley rats (400–450 g) were obtained from the Experimental Animal Center of Fujian Medical University. Animals were housed under controlled conditions (temperature: 18–26 °C, humidity: 40–70%, 12-h light/dark cycle) with free access to food and water, and acclimatized for one week before experimentation. Rats were randomly allocated to DHCA or sham-operated control groups (*n* = 6 per group) using a random number table. This sample size was determined to be sufficient by power analysis (*α* = 0.05, power = 80%) based on pilot data. To minimize confounding, the sequence of experimental procedures was also randomized using a random number table for each group.

After 6 h of fasting, anesthesia was induced with sevoflurane. Animals were then restrained, orally intubated using a 14 G trocar, and mechanically ventilated (initial settings: respiratory rate 70 breaths/min, tidal volume 8–10 mL/kg). Anesthesia was maintained with 3% sevoflurane in pure oxygen. Ventilation was paused during CPB and resumed after weaning, with parameters adjusted according to blood gas measurements throughout the procedure. Rectal temperature was monitored with a paraffin-lubricated electronic thermometer inserted 5 cm into the anus. A multistage venous drain tube (4.5 mm diameter, 50 cm length) was introduced *via* the right external jugular vein into the inferior vena cava and right atrium to establish venous outflow. Systemic heparinization was achieved with 200 IU heparin after cannulation. A 20-G cannula was placed in the caudal artery as the arterial perfusion line, and mean arterial pressure was monitored *via* a 22-G needle in the left femoral artery. All cannulated vessels were ligated distally with silk threads, which also secured the proximal cannula to prevent dislodgement. The CPB circuit consisted of an external jugular vein cannula, a venous reservoir, a roller pump (Stockert III, Germany), a heat exchanger (Xi Jing, China), a temperature-regulating water bank (Stockert III, Germany), an oxygenator (Xi Jing, China; comprising two acrylic sheets encasing a disposable three-layer hollow-fiber membrane), and an arterial line. Before use, the circuit was primed and de-aired with 10 mL of multiple electrolytes injection (Jiameina, China) and 5 mL of 6% hydroxyethyl starch 130/0.4 (Volulyte, China).

**Figure 1.**
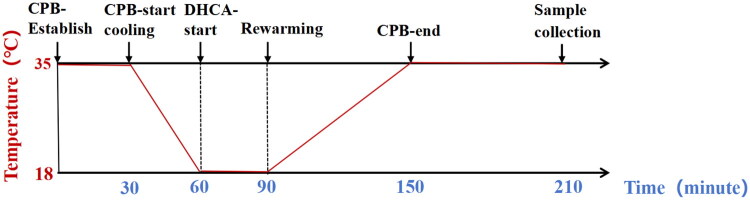
The DHCA model schematic. CPB: cardiopulmonary bypass; DHCA: deep hypothermic circulatory arrest.

Following cannulation, the CPB circuit was connected and bypass initiated. The initial flow rate was set at 120–160 mL/kg/min (approximating physiological rat cardiac output) and was gradually adjusted (by one-half to one-third) during cooling and rewarming phases to maintain mean arterial pressure >40 mmHg for adequate organ perfusion. After 3 min of normothermic CPB, whole-body cooling was initiated using a heat exchanger and temperature-regulated water bath, supplemented by local cerebral cooling with ice packs. This protocol reduced rectal temperature to 18 °C within 30 min, as previously established [6]. At this point, CPB and ventilator support were stopped to induce total circulatory arrest for 30 min. CPB was then resumed, and rewarming was conducted over ∼60 min until rectal temperature reached 35 °C, after which CPB was discontinued. The total CPB duration was about 150 min. Ventilator support was continued for 1 h post-weaning. Any rat exhibiting cardiorespiratory arrest during the experiment was removed from the study protocol. Then the blood samples were collected by left ventricular puncture, and kidney tissues were harvested. A portion of the tissue was snap-frozen in liquid nitrogen, while the remainder was fixed in 4% paraformaldehyde for pathological analysis. Thereafter, rats were euthanized with an overdose of sodium pentobarbital (100 mg/kg). Sham-operated rats underwent anesthesia, intubation, and heparinization for 210 min, followed by the same sample-collection procedures. The DHCA model schematic is shown in Figure 1 and Supplementary Figure S1.

### 2.2. RNA extraction and library preparation

Kidney tissues were collected immediately after DHCA. Total RNA was extracted from 60 mg of tissue using Trizol reagent (Invitrogen, USA). RNA quality and quantity were assessed with a NanoDrop spectrophotometer and an Agilent 2100 Bioanalyzer (Thermo Fisher Scientific, USA); all samples met quality criteria (260/280 ratio > 2.0, RIN > 7). cDNA libraries were prepared from 1 µg of total RNA using the Illumina TruSeq RNA kit and sequenced on an Illumina NovaSeq 6000 platform (Tsingke, China).

### 2.3. Transcriptome analysis

Raw sequencing data quality was assessed with FastQC, followed by trimming and filtering using Trimmomatic. Clean reads were aligned to the rat reference genome (Ensembl 107) with HISAT2, and gene counts were quantified by StringTie. Differentially expressed genes (DEGs) were identified using DESeq2, limma, and edgeR, with thresholds of adjusted *p* < 0.01 and |log_2_FC| ≥ 2. DEGs were visualized as heatmaps and volcano plots using the R packages pheatmap and ggplot2. The intersection of DEGs from all three methods was taken for downstream analysis.

### 2.4. Weighted gene co-expression network analysis (WGCNA)

WGCNA was performed to identify co-expression modules associated with DHCA. Genes with standard deviation >0.5 across samples were retained. After sample clustering and outlier removal, a scale-free network was constructed using a one-step algorithm with a soft-thresholding power (*β* = 7) determined by the pickSoftThreshold function. The adjacency matrix was converted into a topological overlap matrix (TOM), and hierarchical clustering with dynamic tree-cutting (minimum module size = 60 genes) was applied to define modules. Gene significance and module membership were calculated to identify trait-related modules. Network visualization was generated using heatmaps. All analyses were conducted with custom R scripts provided in the Supplementary Materials.

### 2.5. Functional enrichment analysis

Candidate hub genes were defined as the intersection of DEGs and genes from the most significant WGCNA module. Gene Ontology (GO) and Kyoto Encyclopedia of Genes and Genomes (KEGG) pathway enrichment analyses were performed on these genes using the clusterProfiler R package. GO analysis covered biological processes (BP), cellular components (CC), and molecular functions (MF). An adjusted *p*-value <0.05 was set as the significance threshold for enrichment.

### 2.6. PPI network construction and hub gene identification

A PPI network for the candidate hub genes was built using the STRING database, with interactions above a combined score of 0.4 considered significant. The network was visualized in Cytoscape, and the CytoHubba plugin was used to identify the top 10 hub genes based on connectivity. mRNA expression levels of these hub genes were compared between the DHCA and control groups. Their predictive value for DHCA-AKI risk was assessed using receiver operating characteristic (ROC) curves, with an area under the curve (AUC) ≥0.7 considered clinically relevant.

### 2.7. Identification of upstream regulators

Upstream transcription factors (TFs) regulating the hub genes were predicted using the iRegulon plugin (v1.3) in Cytoscape [7]. The analysis integrated 9713 position weight matrices and 1120 ENCODE ChIP-seq tracks, scanning regions ±20 kb from transcription start sites. Stringent thresholds were applied: normalized enrichment score (NES) ≥ 3, motif similarity FDR ≤ 0.001, and AUC ≥ 0.03. Significantly enriched TFs and their target genes were identified by cross-referencing motif enrichment with ChIP-seq signals, followed by functional association analysis.

### 2.8. Comparison with other AKI models

To delineate molecular specificity, DEGs from DHCA-AKI were compared with those from renal ischemia-reperfusion injury (IRI-AKI) and sepsis-associated AKI (SA-AKI). Public kidney transcriptome datasets for mouse IRI (GSE283374) and SA-AKI (GSE256430) models were obtained from GEO database. DEGs in each dataset were called using limma (adjusted *p* < 0.01, |log_2_FC| ≥ 2), and the overlap as well as distinctions among the three AKI types were analyzed.

### 2.9. In vivo *experimental verification*

#### 2.9.1. Biochemical assays

Serum creatinine (Cr, sarcosine oxidase method; mlbio, M12C4B), blood urea nitrogen (BUN, spectrophotometric method; mlbio, ml076478), and NGAL (rat ELISA kit; mlbio, ml003302V) were measured according to the manufacturers’ protocols.

#### 2.9.2. Quantitative RT-PCR

Total RNA was extracted with the RNeasy Mini Kit (Qiagen, 74104), reverse-transcribed using PrimeScript™ RT Master Mix (Takara, RR036A), and amplified with TB Green^®^ Premix Ex Taq™ II (Takara, RR820A). β-actin served as the reference gene. Primers for the ten rat hub genes are listed in Supplementary Table S1. Relative mRNA levels were calculated by the 2 − ΔΔCt method.

#### 2.9.3. Histology and immunohistochemistry

Paraffin-embedded kidney sections (4 µm) were deparaffinized, rehydrated, and stained with hematoxylin & eosin (H&E) or periodic acid-Schiff (PAS) using standard protocols. For immunohistochemistry (IHC), antigen retrieval was performed in citrate buffer (pH 6.0). Endogenous peroxidase was blocked with 3% H_2_O_2_, followed by blocking with normal goat serum. Sections were incubated overnight at 4 °C with primary antibodies against the ten hub proteins (detailed in Supplementary Table S2), then with HRP-conjugated secondary antibody (Cell Signaling Technology, 7074) and developed with DAB. The histological injury score based on H&E staining was determined by summing the scores assigned to the following features: normal histology (1 point), cellular degeneration or nuclear pyknosis (1 point), inflammatory cell infiltration (1 point), and cell necrosis with brush border loss or epithelial cell detachment (2 points). The histological injury score based on PAS staining was calculated by summing the scores of the following criteria: normal morphology (1 point), tubular basement membrane disruption (1 point), and glomerular mesangial thickening (1 point). IHC scores (0–3) were multiplied by the percentage of positive cells to obtain a final score.

#### 2.9.4. Western blot

Proteins were extracted with a phosphoprotein extraction kit (KGI Bio, KGP9100), quantified by BCA assay (Beyotime, P0010S), separated by SDS-PAGE, and transferred to PVDF membranes. After blocking, membranes were incubated overnight at 4 °C with primary antibodies against JUN, ATF3, IL-1β, CXCL1, NFκBIZ, TNFAIP3, and β-actin (details in Supplementary Table S2), followed by HRP-conjugated secondary antibodies (Cell Signaling Technology, 7074/7076) and chemiluminescent detection.

### 2.10. In vitro *cell-based validation*

#### 2.10.1. Cell culture and treatment

Human Kidney-2 (HK-2) cells (Procell, CL-0109) were cultured in complete medium (Procell, CM-0109) at 37 °C with 5% CO_2_, with medium changes every 2–3 days. At 70–80% confluence, cells in the DHCA group were switched to sugar-free DMEM (Solarbio, D6540) and transferred to a hypoxia chamber (DWS H35, UK) set at 1% O_2_, 5% CO_2_, 94% N_2_ and 18 °C for 2 h, followed by return to normoxic conditions (37 °C, 5% CO_2_) for 1 h to simulate reperfusion. Control cells were maintained under normoxia.

#### 2.10.2. Immunofluorescence (IF)

After treatment, cells were washed with PBS, fixed in 4% paraformaldehyde, permeabilized with 0.5% Triton X-100, and blocked with QuickBlock™ buffer. Incubation with primary antibodies (listed in Supplementary Table S2) was performed overnight at 4 °C. After washing, cells were incubated with Alexa Fluor^®^ 488-conjugated anti-rabbit IgG (Cell Signaling Technology, 4412) for 1 h in the dark. Nuclei were counterstained with DAPI-containing mounting medium, and images were captured using a fluorescence microscope.

#### 2.10.3. Quantitative RT-PCR

Total RNA was extracted from HK-2 cells, reverse-transcribed, and amplified as described in Section 2.9. Primer sequences for the ten human orthologs of the hub genes are provided in Supplementary Table S3.

### 2.11. Statistical analyses

Outcome assessors and data analysts remained blinded to group allocation and the specific experimental procedures. All statistical analyses were performed using R language (Version 4.3.3) and GraphPad Prism software (Version 9.5.0). Data distribution and variance homogeneity were assessed using Shapiro-Wilk and Levene’s tests, respectively. The *t*-test was applied when assumptions were met; otherwise, the Wilcoxon test was employed. A significance level of *p* < 0.05 was considered indicative of a statistically significant difference.

## 3. Results

### 3.1. Model construction

The DHCA model was successfully established in all rats, with hematocrit sustained at the target level of ∼20% throughout the DHCA period [8]. For transcriptome sequencing, three biological replicates per group (DHCA and control) were used to meet minimum sample requirements [6]. To mitigate potential bias from the modest sample size, sequencing depth was increased for each individual sample. Key differential expression findings were subsequently subjected to experimental validation.

### 3.2. Identification of DEGs

**Figure 2.**
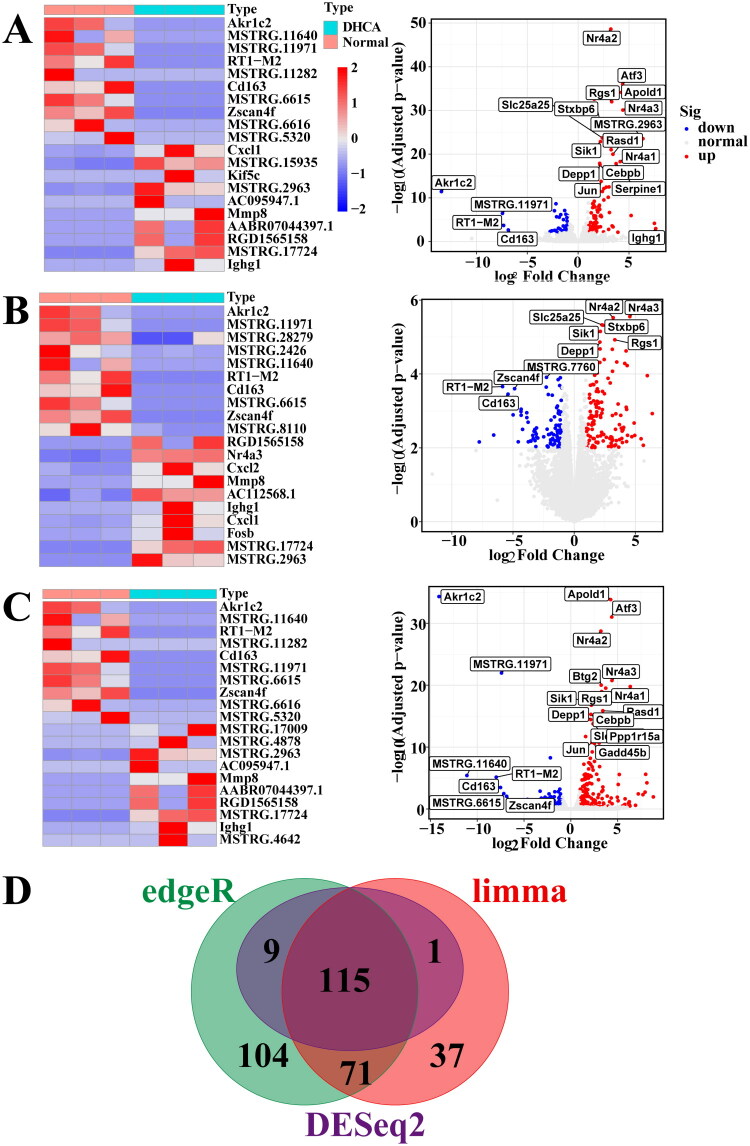
Identification of differentially expressed genes (DEGs). (A–C) Heatmaps and volcano plots of DEGs identified by DESeq2 (A), limma (B), and edgeR (C). (D) Venn diagram of DEGs overlapping across all three methods. DHCA: deep hypothermic circulatory arrest.

Kidney tissues were collected 1 h after CPB weaning for transcriptome sequencing (DHCA: *n* = 3; control: *n* = 3). Of 25,507 detected transcripts, 19,012 were mapped to the rat genome. Differential expression analysis using DESeq2, limma, and edgeR identified 125, 224, and 299 DEGs, respectively (Figures 2(A–C)). The intersection of these three gene sets yielded 115 robust DEGs (77 up-regulated, 38 down-regulated; Figure 2(D)).

### 3.3. Construction of the co-expression network

**Figure 3.**
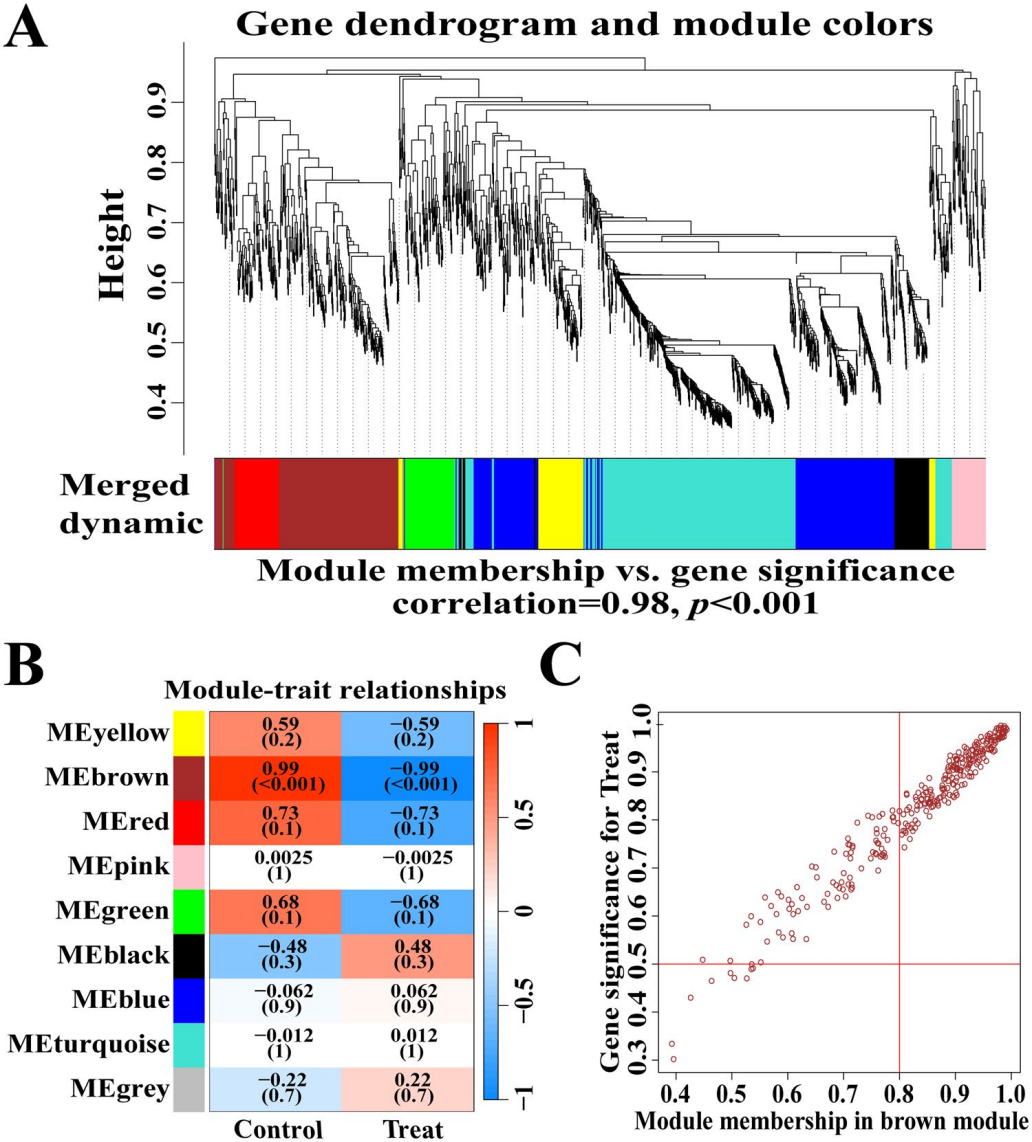
WGCNA identifies key modules associated with DHCA. (A) Dendrogram illustrating the clustering of all genes based on the topological overlap matrix (1-TOM). (B) Module-trait heatmap displaying clustered gene modules along with their correlation to renal function post-DHCA, including corresponding correlation coefficients and *p*-values for each module. (C) Scatter plot indicating a significant positive correlation between the brown module and renal function post-DHCA. WGCNA: weighted gene co-expression network analysis; TOM: topological overlap matrix; DHCA: deep hypothermic circulatory arrest.

WGCNA analysis was conducted on genes derived from transcriptome sequencing across all samples to evaluate gene expression associated with DHCA-AKI (Figure 3(A)). A total of nine distinct modules were identified (Figure 3(B)). Through the analysis of positive correlation coefficients, we found a highly significant association between the brown module and DHCA-AKI (correlation coefficient = 0.98, *p* < 0.001, Figure 3(C)), which included 209 core genes.

### 3.4. Functional enrichment analysis of candidate hub genes

**Figure 4.**
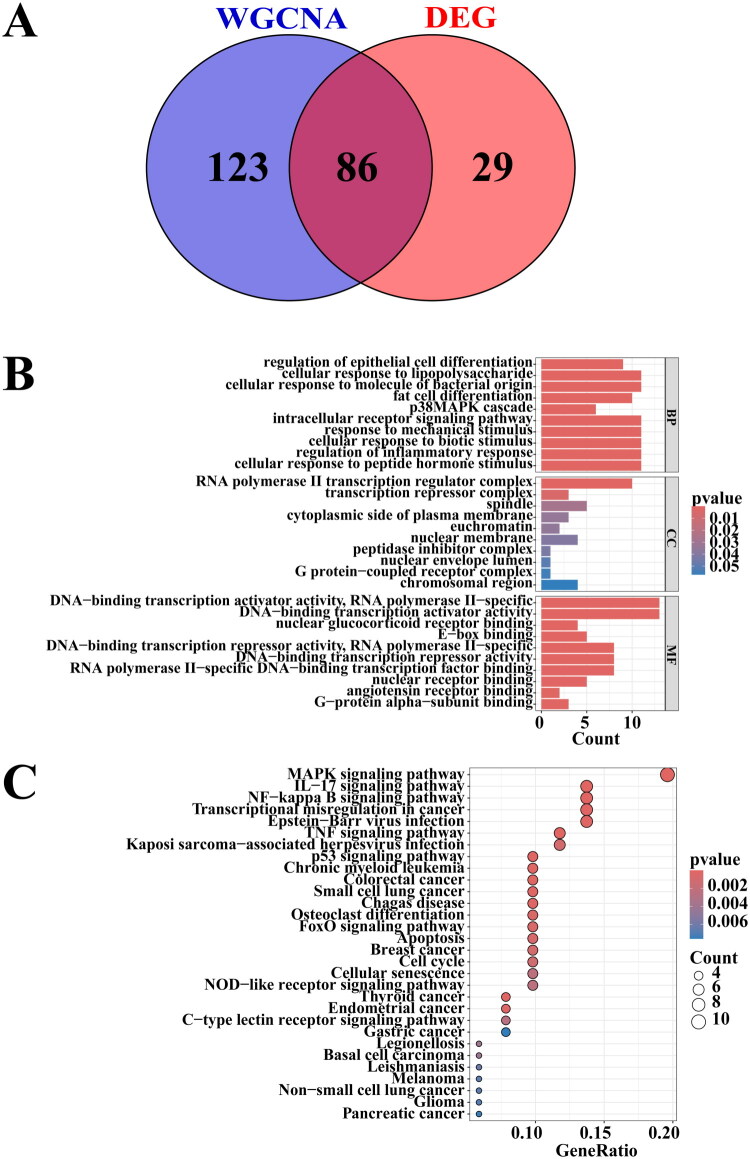
Screening and functional enrichment analysis of candidate hub genes. (A) The Venn diagram illustrated the identification of 86 candidate hub genes derived from the intersection of WGCNA and DEGs. (B) GO enrichment analysis of the candidate hub genes. (C) KEGG pathway analysis for the candidate hub genes. GO: Gene Ontology; KEGG: Kyoto Encyclopedia of Genes and Genomes; WGCNA: weighted gene co-expression network analysis; DEGs: differentially expressed genes.

The 209 genes from the DHCA-associated brown module were intersected with the 115 DEGs, yielding 86 candidate hub genes (Figure 4(A)) considered central to DHCA-AKI pathogenesis. GO enrichment analysis showed these 86 genes were primarily involved in: biological processes such as response to lipopolysaccharide/bacterial molecules, inflammatory regulation, and p38 MAPK cascade; cellular components including transcriptional regulatory complexes and G-protein-coupled receptor complexes; and molecular functions like DNA-binding transcription factor activity and nuclear receptor binding (Figure 4(B)). KEGG pathway analysis further linked the candidate genes to key signaling pathways: MAPK, IL-17, NFκB, TNF, p53, FoxO, apoptosis, NOD-like receptor, and C-type lectin receptor pathways (Figure 4(C)).

### 3.5. PPI network construction and hub gene identification

**Figure 5.**
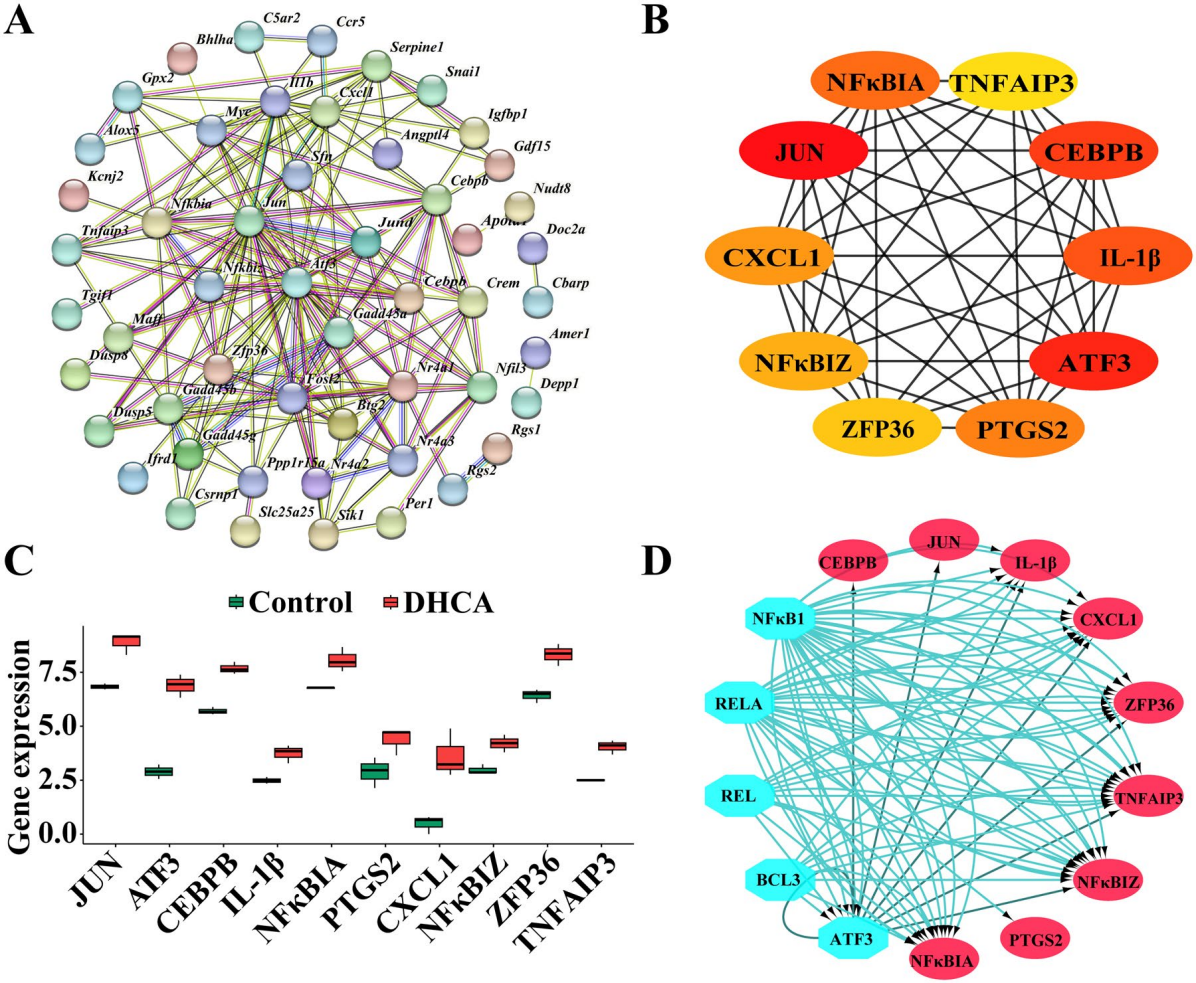
Protein–protein interaction (PPI) network and hub gene identification. (A) PPI network of candidate hub genes. (B) Top 10 hub genes within the PPI network. (C) mRNA expression levels of the 10 hub genes in DHCA *vs.* control groups. (D) Upstream transcription factors predicted by iRegulon. DHCA: deep hypothermic circulatory arrest.

A PPI network was generated from the 86 candidate genes using STRING (Figure 5(A)). The CytoHubba plugin in Cytoscape identified the top 10 hub genes (Figure 5(B)): *Jun, Atf3, Cebpb, Il-1β, Nfκbia, Ptgs2, Cxcl1, Nfκbiz, Zfp36, and Tnfaip3*. Node color intensity corresponds to connectivity within the network, with *Jun* displaying the highest score. These hub genes were significantly upregulated in the DHCA group at the mRNA level (Figure 5(C)). ROC analysis showed an AUC of 1.000 for each gene, indicating perfect predictive ability for DHCA-AKI (Supplementary Figure S2).

### 3.6. Identification of upstream regulators of hub genes

A gene regulatory network was constructed, comprising 14 hub genes, among which 5 were identified as upstream regulators. RELA and ATF3 exhibited the most significant results, achieving NEScores of 8.922 and 6.317, respectively. RELA regulated eight target genes, including ATF3, IL-1β, NFκBIA, PTGS2, CXCL1, NFκBIZ, ZFP36, and TNFAIP3. ATF3 regulated all ten hub gene targets (Figure 5(D)).

### 3.7. Comparison with other AKI types

Comparison of DEGs revealed distinct molecular profiles for DHCA-AKI. While the renal IRI model (GSE283374; 613 DEGs) shared only three genes (*Atf3*, *Ptgs2*, *Nfκbiz*) with DHCA-AKI, and the SA-AKI model (GSE256430; 387 DEGs) shared only two (*Atf3*, *Tnfaip3*) (Supplementary Figure S3), these limited overlaps suggest DHCA-AKI possesses a unique transcriptional signature. The overlapping genes may represent common pathogenic mechanisms across AKI subtypes.

### 3.8. Assessment of kidney function

**Figure 6.**
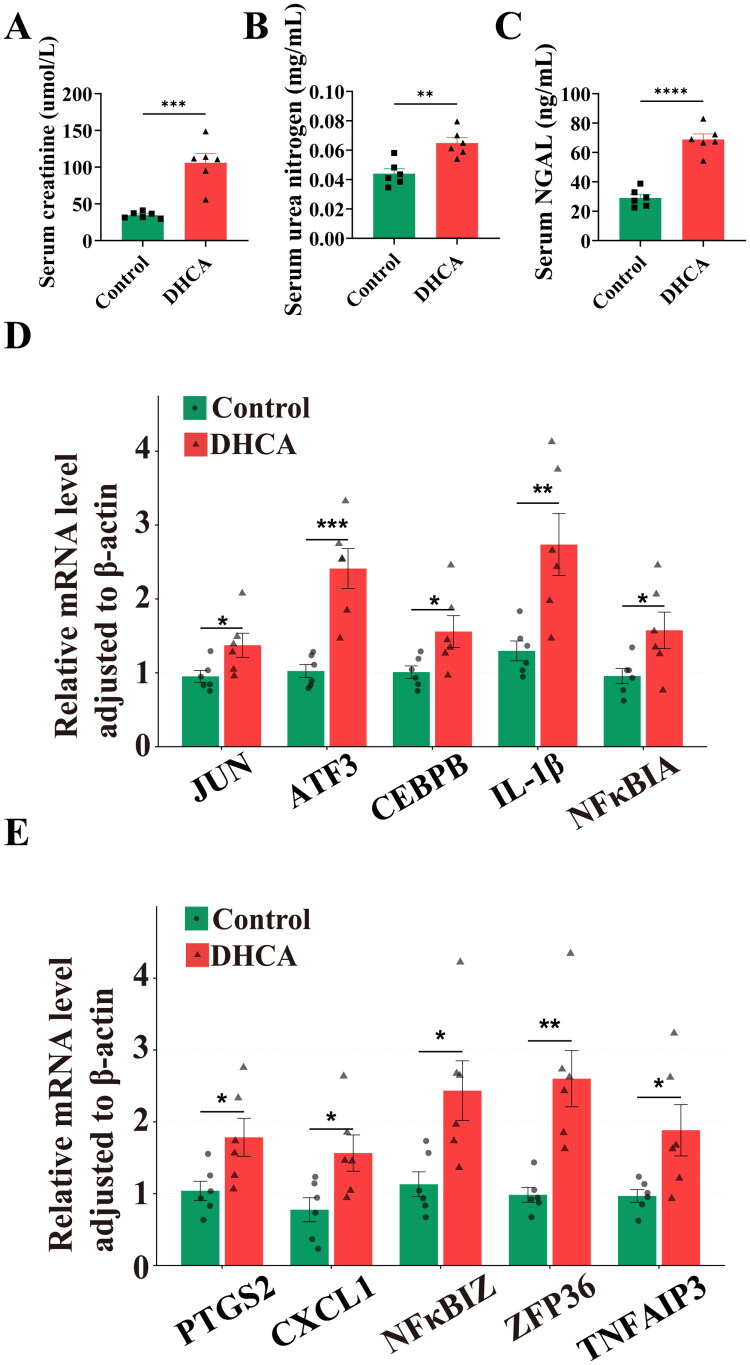
Functional and molecular comparison between rat DHCA and control groups. (A) Serum creatinine (Cr). (B) Blood urea nitrogen (BUN). (C) Serum neutrophil gelatinase‑associated lipocalin (NGAL). (D, E) mRNA expression of the ten hub genes. DHCA: deep hypothermic circulatory arrest. *p < 0.05, **p < 0.01, ***p < 0.001, ^****^p < 0.0001.

Serum levels of Cr, BUN, and NGAL were significantly elevated in the DHCA group compared with the control group (*p* < 0.01; Figures 6(A–C)), indicating substantial impairment of kidney function.

### 3.9. Hub gene expression in kidney tissues

RT-PCR analysis confirmed that all ten hub genes were significantly upregulated at the mRNA level in DHCA kidneys compared to controls (Figures 6(D,E)).

### 3.10. Kidney histopathology and hub gene protein expression

**Figure 7.**
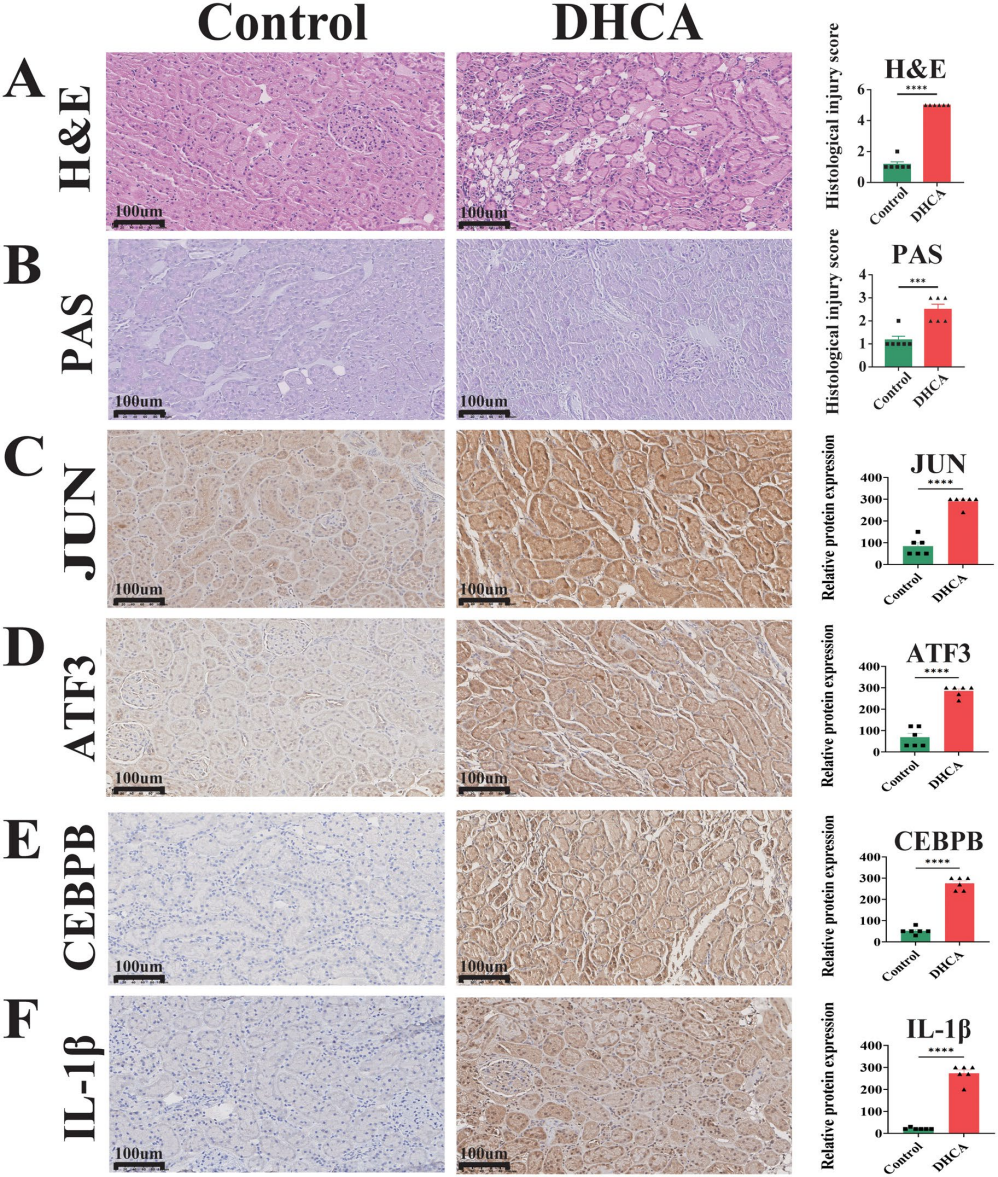
Histological assessment of renal injury and hub gene expression. (A) H&E staining of kidney tissue. (B) PAS staining of kidney tissue. (C–F) IHC staining for JUN, ATF3, CEBPB, and IL‑1β in renal sections from DHCA and control groups. HE: hematoxylin and eosin; PAS: periodic acid-Schiff; IHC: immunohistochemistry; DHCA: deep hypothermic circulatory arrest. ***p < 0.001, ^****^p < 0.0001.

**Figure 8.**
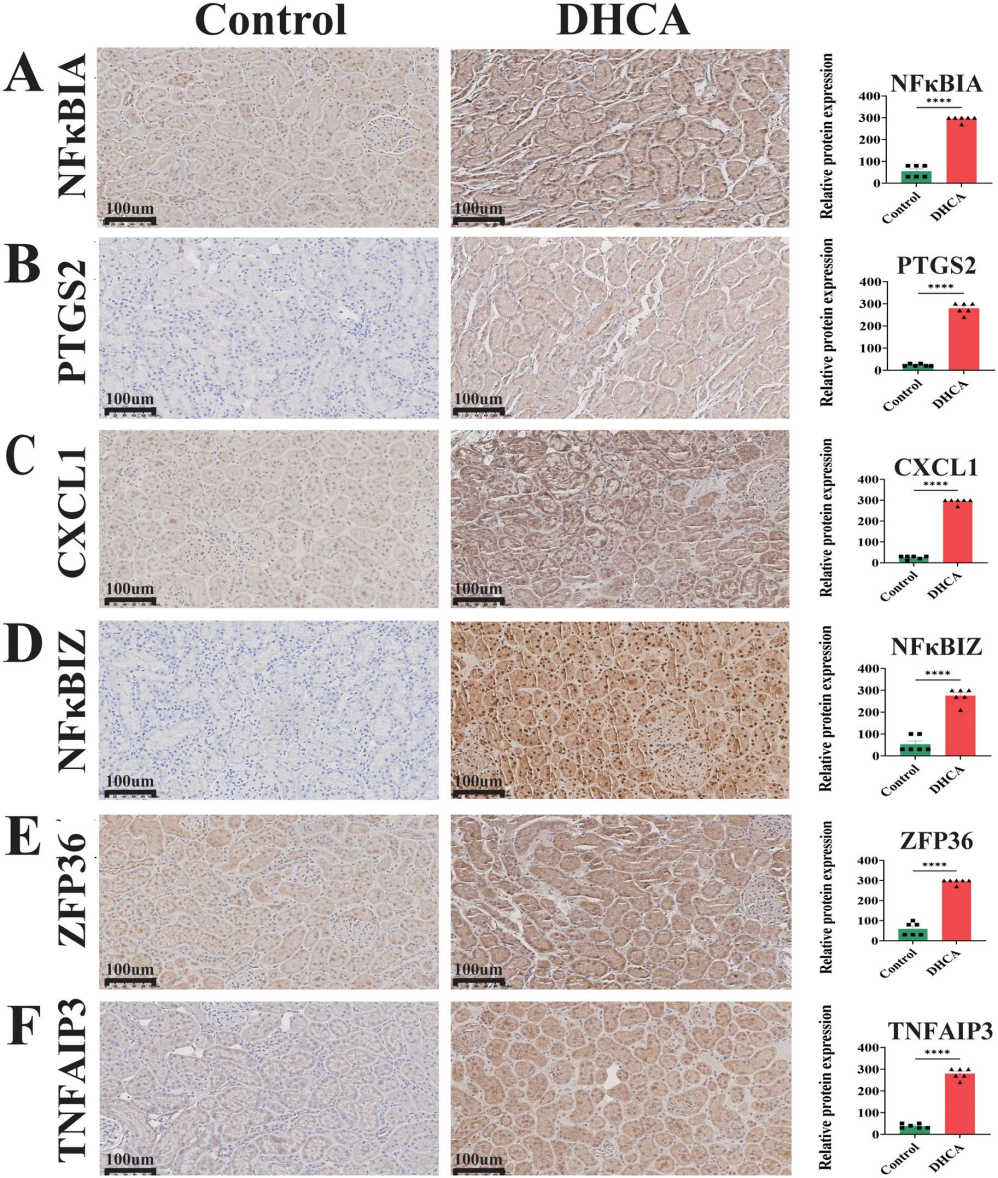
Immunohistochemical analysis of hub gene expression. (A–F) IHC staining for NFκBIA, PTGS2, CXCL1, NFκBIZ, ZFP36, and TNFAIP3 in kidney tissues from DHCA and control groups. IHC: immunohistochemistry; DHCA: deep hypothermic circulatory arrest. ^****^p < 0.0001.

Histological assessment by H&E and PAS staining revealed significantly greater histological injury in the DHCA group, characterized by cellular degeneration or nuclear pyknosis, inflammatory cell infiltration, brush border loss, epithelial cell detachment, and tubular basement membrane disruption (Figures 7(A,B)), consistent with activation of apoptotic pathways linked to the hub genes. IHC further demonstrated strong upregulation of all ten hub gene proteins in DHCA kidneys, with intense positive staining compared to weak or negative signals in controls (Figures 7(C–F) and 8(A–F)), confirming their significant induction in response to DHCA.

### 3.11. Western blot analysis

**Figure 9.**
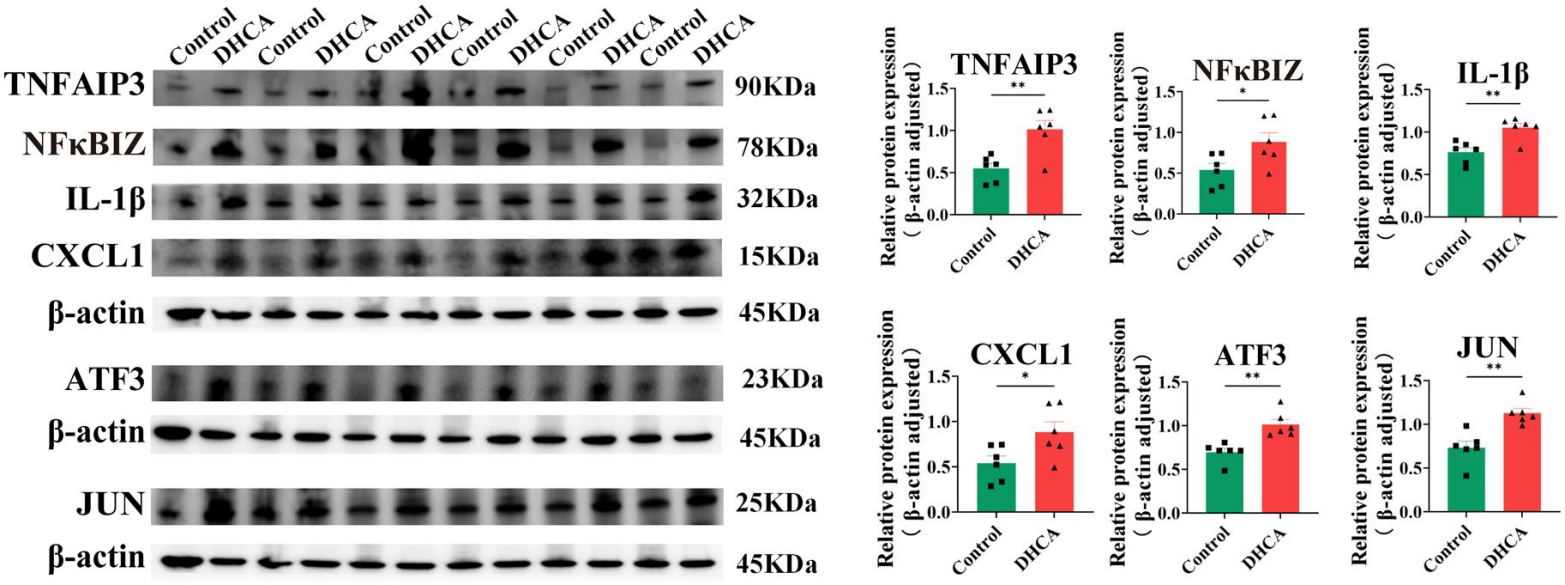
Western blot analysis of hub gene expression in DHCA and control groups. DHCA: deep hypothermic circulatory arrest. *p < 0.05, **p < 0.01.

Western blot analysis confirmed elevated protein levels of JUN, ATF3, IL-1β, CXCL1, NFκBIZ, and TNFAIP3 in DHCA kidneys compared to controls (Figure 9), corroborating the upregulation of these hub genes.

### 3.12. Immunofluorescence staining in HK-2 cells

**Figure 10.**
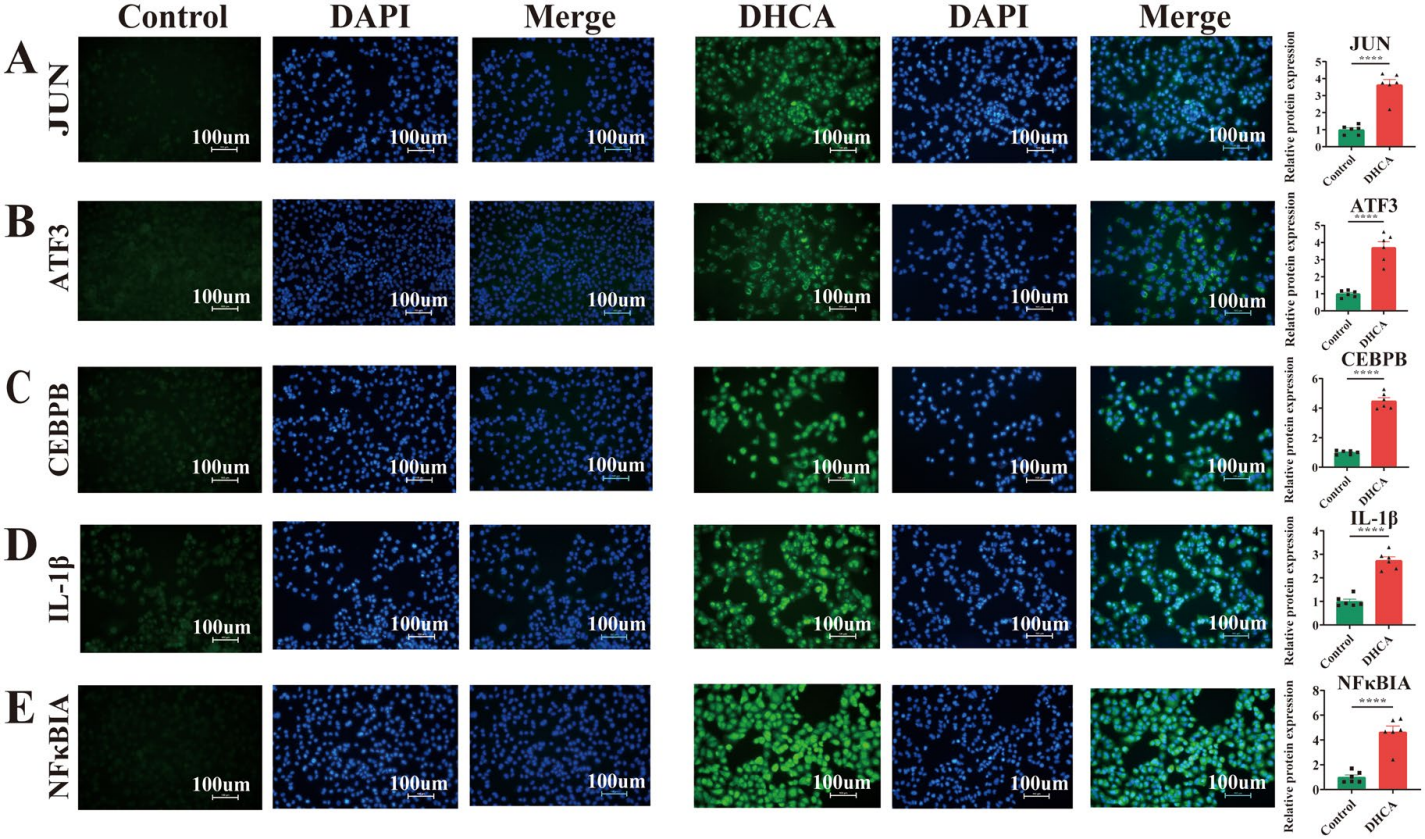
Immunofluorescence of hub genes in HK-2 cells in DHCA and Control groups. (A–E) Comparison of IF for JUN, ATF3, CEBPB, IL-1β, and NFκBIA between the two groups. DHCA: deep hypothermic circulatory arrest. ^****^p < 0.0001.

**Figure 11.**
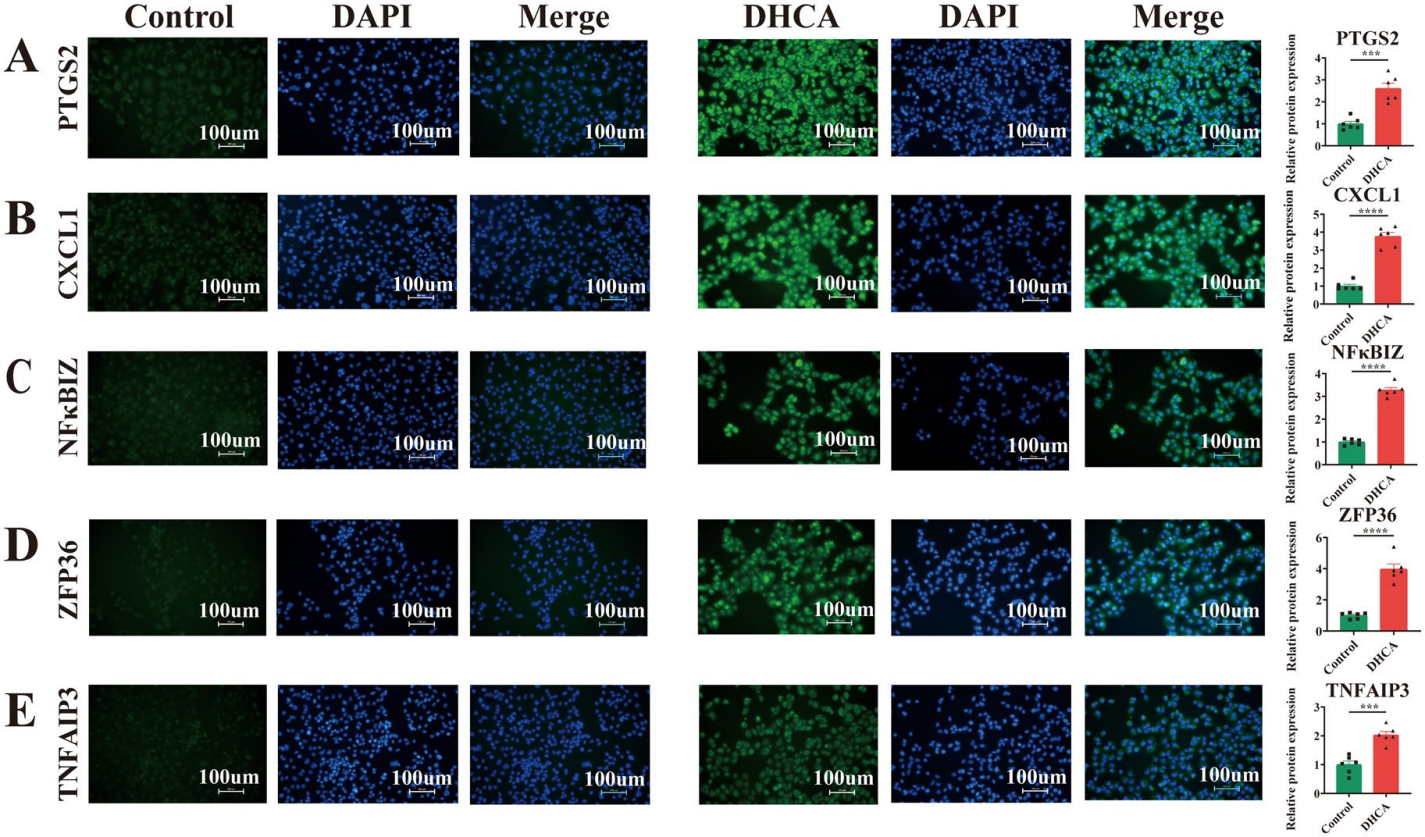
Immunofluorescence of hub genes in HK-2 cells in DHCA and Control groups. (A–E) Comparison of IF for PTGS2, CXCL1, NFκBIZ, ZFP36, and TNFAIP3 between the two groups. DHCA: deep hypothermic circulatory arrest. ***p < 0.001, ^****^p < 0.0001.

Immunofluorescence staining in HK-2 cells subjected to simulated DHCA showed significantly higher protein expression of all ten hub genes relative to normoxic controls (Figures 10 and 11).

### 3.13. Hub gene expression in HK-2 cells

**Figure 12.**
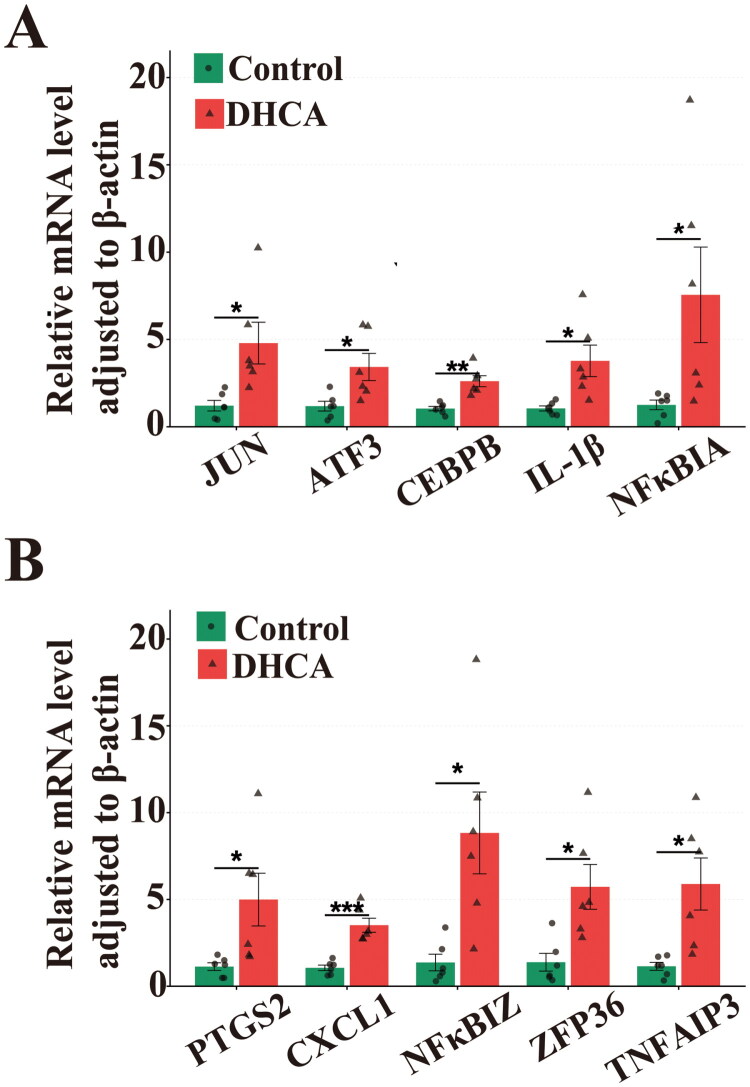
Comparison of mRNA levels of hub genes between the two HK-2 groups. (A) Comparison of mRNA expression levels of JUN, ATF3, CEBPB, IL-1β, and NFκBIA between the two HK-2 groups. (B) Comparison of mRNA expression levels of PTGS2, CXCL1, NFκBIZ, ZFP36, and TNFAIP3 between the two HK-2 groups. DHCA: deep hypothermic circulatory arrest. *p < 0.05, **p < 0.01, ***p < 0.001.

Consistent with tissue findings, RT-PCR demonstrated marked upregulation of all ten hub genes at the mRNA level in HK-2 cells under DHCA-mimetic conditions (Figure 12).

## 4. Discussion

In this study, we identified ten hub genes—*Jun*, *Atf3*, *Cebpb*, *Il-1β*, *Nfκbia*, *Ptgs2*, *Cxcl1*, *Nfκbiz*, *Zfp36*, and *Tnfaip3*—that were significantly altered in rat kidneys following DHCA. These genes were pinpointed through integrated transcriptomic sequencing, differential expression analysis, WGCNA, and PPI mapping, and are predominantly linked to inflammatory and apoptotic pathways. Experimental validation confirmed substantial kidney injury in the DHCA group, accompanied by marked upregulation of these hub genes. Our findings address an important knowledge gap by delineating key transcriptomic changes and associated molecular pathways in DHCA-AKI.

To our knowledge, this is the first report detailing the transcriptional alterations in kidney tissues induced by DHCA. It is important to emphasize that DHCA-AKI differs fundamentally from IRI-AKI in both clinical context and pathophysiology. IRI-AKI typically results from warm ischemia during events such as kidney transplantation or hemorrhagic shock, followed by reperfusion-driven oxidative stress and inflammation [7]. In contrast, DHCA-AKI arises in the setting of aortic arch surgery, where total circulatory arrest is combined with deep hypothermia to lower metabolic demand. Despite this protective intent, kidney injury still occurs due to hypothermia-mediated vasoconstriction, endothelial dysfunction, microcirculatory impairment, and reperfusion injury upon rewarming. Moreover, DHCA is accompanied by systemic inflammation, hemolysis, and elevated free hemoglobin related to CPB [9]. Experimentally, IRI-AKI is commonly modeled by renal artery clamping or hypoxia-reoxygenation *in vitro*, whereas DHCA-AKI requires a complex model integrating CPB and circulatory arrest under deep hypothermia. Although both involve ischemia, DHCA imposes additional burdens from hypothermia itself, multi-organ crosstalk, and CPB-associated inflammatory cascades. Our comparative transcriptomic analysis revealed many non-overlapping DEG profiles between DHCA-AKI and IRI-AKI, underscoring that DHCA initiates a distinct and clinically specific pathophysiological process.

Our findings demonstrate rapid renal functional impairment following DHCA, evidenced by significant elevations in serum Cr, BUN, and NGAL within 2 h. Clinically, serum Cr levels in patients following AD surgery typically begin to rise within hours to days after DHCA, with most cases classified as KDIGO stage 1–2, though some progress to stage 3 [10]. The high metabolic activity of renal tubules, particularly the vulnerable proximal segment, explains the prominent tubular necrosis and apoptosis observed histologically—a hallmark of AKI contributing to filtration dysfunction [11], while glomerular dysfunction may also play a role. The acute kidney injury observed in this study likely stems from the combined insults of deep hypothermia per se, the intense stress of rewarming/reperfusion, and systemic inflammation associated with CPB. These factors collectively drive the early transcriptional activation of stress-responsive, inflammatory, and apoptotic pathways, which aligns with the rapid upregulation of hub gene proteins we observed. This rapid upregulation of hub gene proteins may be a key factor in the pathogenesis of DHCA-AKI.

DHCA is associated with IRI characterized by mitochondrial dysfunction and elevated levels of ROS. ROS can provoke inflammation and oxidative stress by upregulating pro-inflammatory transcription factors such as NFκB [12], leading to cellular damage, cell death, and subsequent development of AKI. The hub genes and pathways identified in our study were predominantly linked to inflammatory responses and cellular apoptosis—findings that align with previous research [3,11,12].

Several studies have utilized animal models of DHCA to investigate kidney function. For instance, Yu et al. demonstrated that cold-inducible ribonucleic acid binding protein (CIRP) exhibits anti-apoptotic and nephroprotective effects during hypothermia treatment, thereby mitigating AKI following DHCA in rats [13]. Furthermore, some study also revealed that the overexpression of miRNA-106b-5p reduced kidney injury after DHCA in rat models [14]. Additionally, a miRNA-targeted database identified differentially expressed miRNAs potentially associated with apoptosis, gene methylation, inflammation, oxidative stress response, and autophagy—all of which are related to ischemic injury [14]. In a DHCA rabbit model, kidney injury was significantly exacerbated, with the rise of elevated serum levels of cystatin C and NGAL [2]. Consequently, genes that are overexpressed in kidney tissues subjected to DHCA may play either protective or pathogenic roles concerning kidney function.

In this study, we identified ten hub DEGs that were significantly elevated in kidney tissues following DHCA. Notably, *Jun* was identified as one of the ten significantly upregulated hub genes in kidneys following DHCA. As a transcription factor (AP-1), JUN is known to promote inflammatory and cytotoxic responses, contributing to kidney injury [15,16]. Its elevated expression in our model suggests that JUN likely exacerbates DHCA-AKI by driving pro-inflammatory signaling and associated cellular damage.

*Atf3*, a stress-inducible gene, was markedly upregulated in DHCA-exposed kidneys. While some studies highlight its protective roles—such as attenuating apoptosis, inflammation, and ROS-mediated injury [17,18]—others suggest it may also promote damage, e.g., by facilitating neutrophil chemotaxis in injured tubules [19]. Furthermore, ATF3 expression was found to be upregulated in mouse models of proteinuria and diabetic nephropathy. *In vitro* experiments demonstrated that podocytes treated with high glucose and lipopolysaccharide exhibited increased apoptosis and injury leading to podocyte loss; these changes ultimately resulted in proteinuria, glomerulosclerosis, and kidney failure [20]. This dual context-dependent function positions ATF3 as a pivotal node in DHCA-AKI pathogenesis and a potential therapeutic target. Notably, our upstream regulatory analysis identified both RELA and ATF3 as key transcriptional regulators of multiple hub genes, further underscoring their central role in the DHCA-AKI network.

The transcription factor *Cebpb* was upregulated in DHCA kidneys and may promote renal injury by driving inflammatory responses and macrophage infiltration—mechanisms supported by its association with lupus nephritis severity and its role in exacerbating IRI *via* miRNA-16 [21,22]. These observations position CEBPB as a potential diagnostic marker and therapeutic target in DHCA-AKI.

*Ptgs2* was upregulated in DHCA kidneys. As an inducible enzyme critical for prostaglandin synthesis in inflammation, PTGS2 can drive inflammatory responses *via* NFκB and AP-1 pathways, as evidenced in preeclampsia, ischemic stroke, and psoriasis models [23–25]. Its elevation in our study suggests that PTGS2 likely contributes to DHCA-AKI by amplifying inflammation through interactions with NFκB, AP-1, and IL-17 signaling.

*Cxcl1*, a neutrophil-chemoattractant chemokine, was elevated in DHCA kidneys. In inflammatory conditions, CXCL1 can amplify IL-6 production *via* CCR2, MAPK, and AP-1 pathways [26], while its downregulation mitigates neuroinflammation after ischemic injury [27]. Thus, upregulation of CXCL1 likely aggravates renal inflammation in DHCA-AKI through interactions with MAPK, AP-1, and TNF-α signaling, contributing to tubular damage and functional decline.

*Nfκbia* and *Nfκbiz*, which encode inhibitors of the NFκB pathway, were upregulated in DHCA kidneys. Their induction may represent a feedback mechanism to restrain inflammation, as suggested by studies showing that suppression of NFκBIA exacerbates cardiomyocyte injury [28] and that variants in NFκBIZ are linked to inflammatory disease risk [29]. Thus, elevated levels of these inhibitors could serve a protective role in mitigating NFκB-driven renal damage following DHCA.

*Zfp36* was upregulated in DHCA kidneys. As an RNA-binding protein, ZFP36 typically exerts protective effects by attenuating inflammation, oxidative stress, and apoptosis—mechanisms demonstrated in models of neuronal injury (*via* NOX4‑DRP1 inhibition) and intestinal ischemia-reperfusion (*via* CREBBP/p53/p21/Bax signaling) [30,31]. Its reduced expression is also linked to inflammatory pathology in psoriasis [32]. Thus, elevated ZFP36 may help limit renal damage during DHCA through similar anti-inflammatory and anti-apoptotic actions.

*Tnfaip3*, a ubiquitin-editing enzyme with potent anti-inflammatory activity, was upregulated in DHCA kidneys. While it exerts neuroprotective effects in epilepsy and Parkinson’s models *via* suppression of NFκB and mTOR pathways [33–35], TNFAIP3 can also promote injury in contexts such as acute pancreatitis through RIP3/NLRP3 activation [36,37]. Its elevated expression in our model suggests a context-dependent role in DHCA-AKI, likely modulating NFκB-driven inflammation with both protective and pathogenic potential.

This study revealed that multiple signaling pathways are potentially involved in DHCA-AKI, primarily centered on inflammation and cell death. Key pathways included NFκB, TNF, IL-17, and p53 (inflammatory response) as well as MAPK, FoxO, and apoptosis (cell proliferation and death). These pathways often interact; for example, FoxO signaling can be regulated by AKT and MAPK. Pharmacological evidence supports their relevance: aprepitant protects against organ injury by suppressing the IGF1/AKT/FOXO1 axis and downstream inflammatory mediators [38], while umbelliferone ameliorates cisplatin-induced nephrotoxicity *via* SIRT1/FOXO-3 and NFκB modulation [39]. Furthermore, NOD-like receptors and C-type lectin receptors (e.g., Mincle) contribute to inflammasome activation and inflammatory cell death in AKI [40], highlighting their potential role in the immune-inflammatory cascade triggered by DHCA.

Therefore, the altered expression of hub genes in DHCA kidneys is directly associated with inflammatory, oxidative, and apoptotic processes in tubular cells. These genes interact within a network linked to key pathways governing inflammation and cell fate. Their collective dysregulation disrupts the balance between injury and repair mechanisms, ultimately driving AKI. Notably, all ten hub genes exhibit strong predictive value for post-DHCA renal dysfunction, highlighting their potential as diagnostic biomarkers for DHCA-AKI.

Collectively, the upregulated hub genes likely promote DHCA-AKI by enhancing tubular apoptosis, amplifying inflammation, disrupting tubular integrity, and reducing glomerular filtration. Among them, the stress-responsive gene ATF3 appears to function as a central regulatory node, capable of activating pro-inflammatory and pro-apoptotic pathways. Thus, ATF3 and other hub genes may serve as early biomarkers for DHCA-AKI, and targeting this regulatory axis could offer a promising therapeutic strategy to improve patient outcomes.

## 5. Limitations

While this study provides the first transcriptomic characterization of DHCA-induced renal injury, several limitations should be noted. The sample size for RNA-seq was modest, though increased sequencing depth and orthogonal validation were used to enhance reliability. The CPB circuit was primed with 6% HES 130/0.4, whose renal impact remains debated; future studies could consider alternative colloids. Glomerular function was not assessed *via* urinary protein, and longitudinal pre-/post-injury samples were not collected—a design chosen to avoid additional procedural trauma, though endpoint differences robustly supported the model.

## 6. Conclusion

Our study reveals that DHCA profoundly alters the renal transcriptome, with inflammation- and apoptosis-related genes and their associated pathways playing central roles in the pathogenesis of DHCA-AKI. These findings provide new mechanistic insights into DHCA-induced kidney injury and highlight promising targets for future renoprotective strategies.

## Supplementary Material

Supplementary FigS1.png

Supplementary FigS2.tif

Supplementary FigS3.tif

Supplementary Materials.docx

## Data Availability

The RNA-seq dataset, immunohistochemistry, Western blot, and immunofluorescence images are accessible on reasonable request *via* the ScienceDB database (DOI: 10.57760/sciencedb.26982).
