# Supplementary figures and images for: Transcriptome and weighted gene co-expression network analysis identify hub genes and pathways in rat kidneys after deep hypothermic circulatory arrest
Source: Ren Fail. 2026 Mar 10;48(1):2635286. doi: 10.1080/0886022X.2026.2635286 (PMC12981254; doi:10.1080/0886022X.2026.2635286)

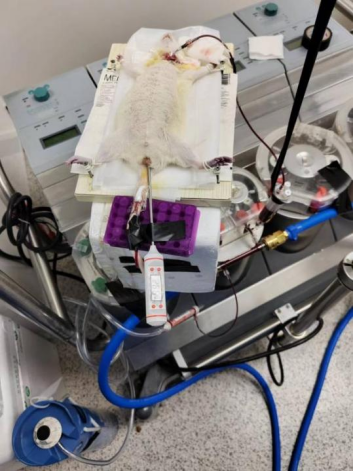

Supplement: Supplementary FigS1.png [file IRNF_A_2635286_SM4656.png]

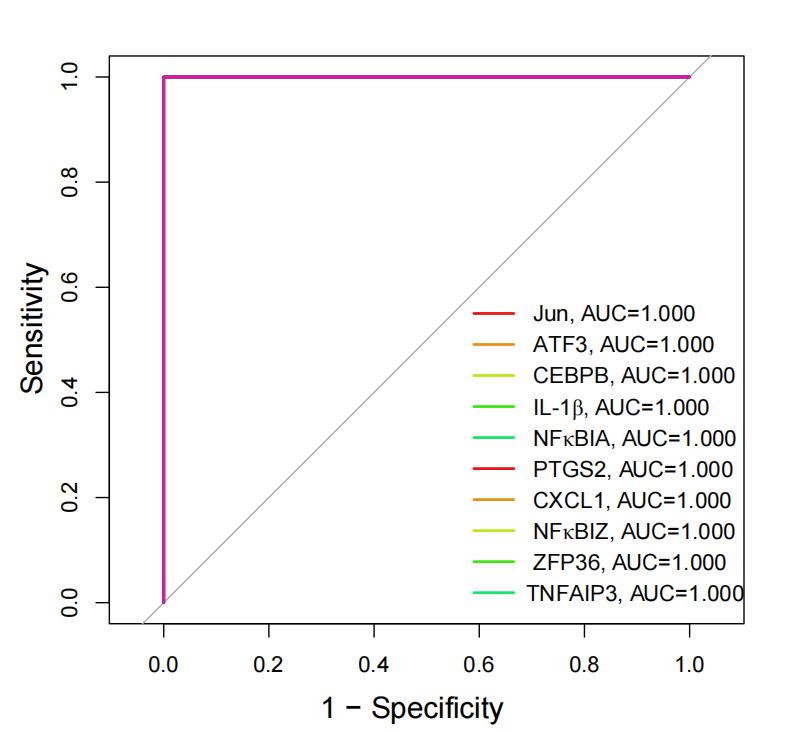

Supplement: Supplementary FigS2.tif [file IRNF_A_2635286_SM4655.tif]

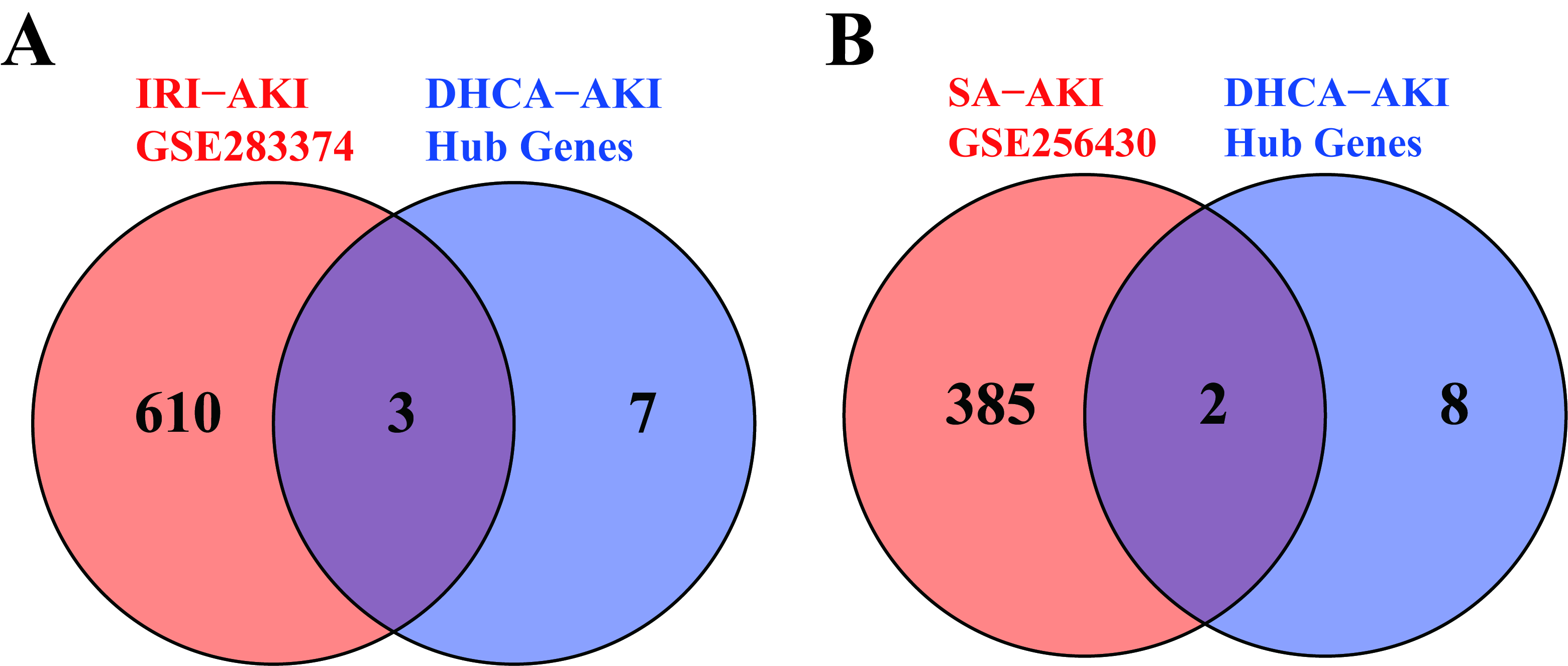

Supplement: Supplementary FigS3.tif [file IRNF_A_2635286_SM4653.tif]
