# Supplementary material for: Transcriptome and weighted gene co-expression network analysis identify hub genes and pathways in rat kidneys after deep hypothermic circulatory arrest
Source: Ren Fail. 2026 Mar 10;48(1):2635286. doi: 10.1080/0886022X.2026.2635286 (PMC12981254; doi:10.1080/0886022X.2026.2635286)
Supplement: Supplementary Materials.docx [file IRNF_A_2635286_SM4652.docx]

#
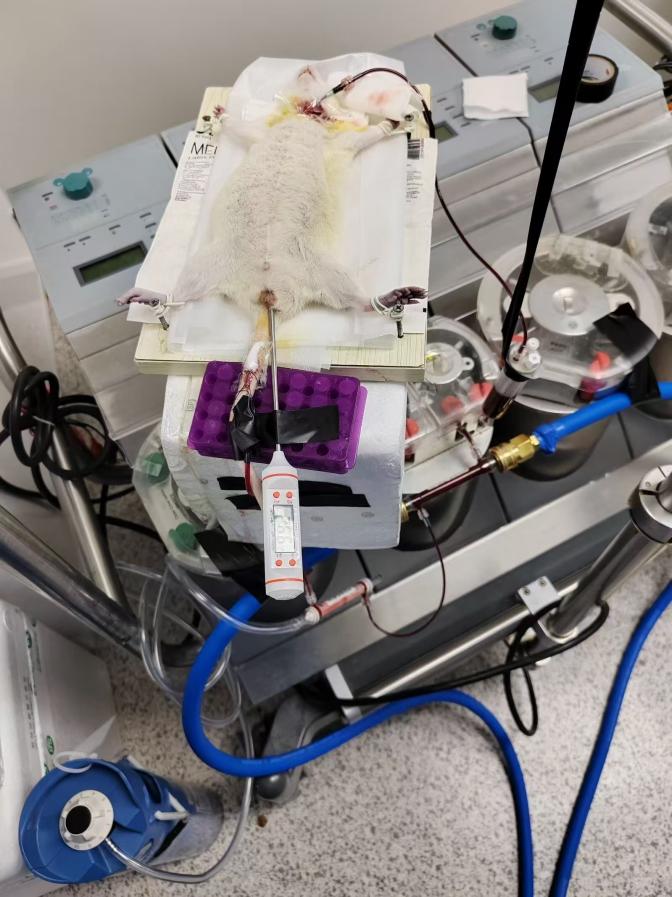


# **Supplementary Fig. S1** Construction of a rat DHCA model. DHCA: deep hypothermic circulatory arrest.

#
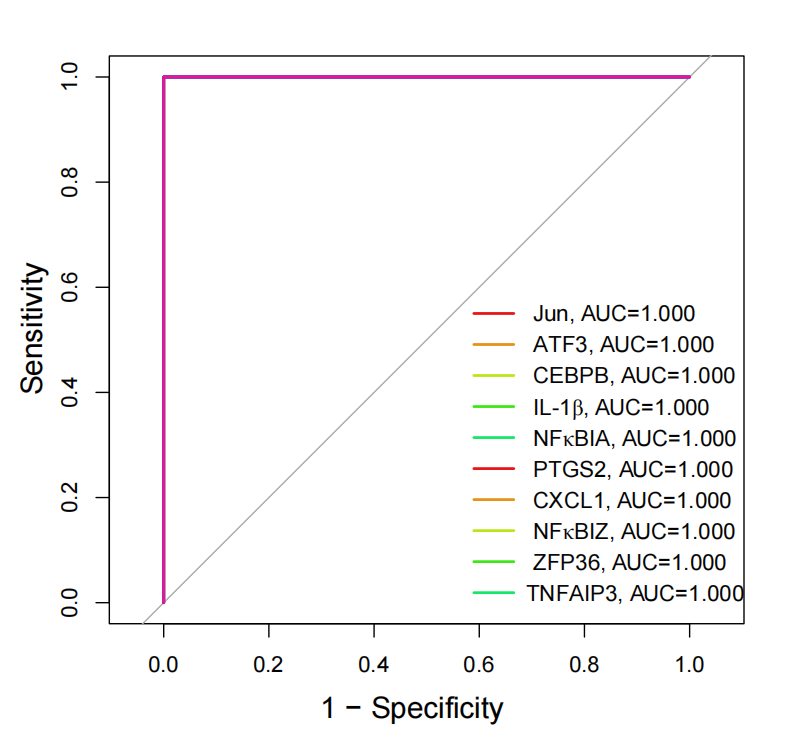


**Supplementary Figure S2** AUC values for each hub genes related to DHCA. AUC: area under the receiver operating characteristic curve.


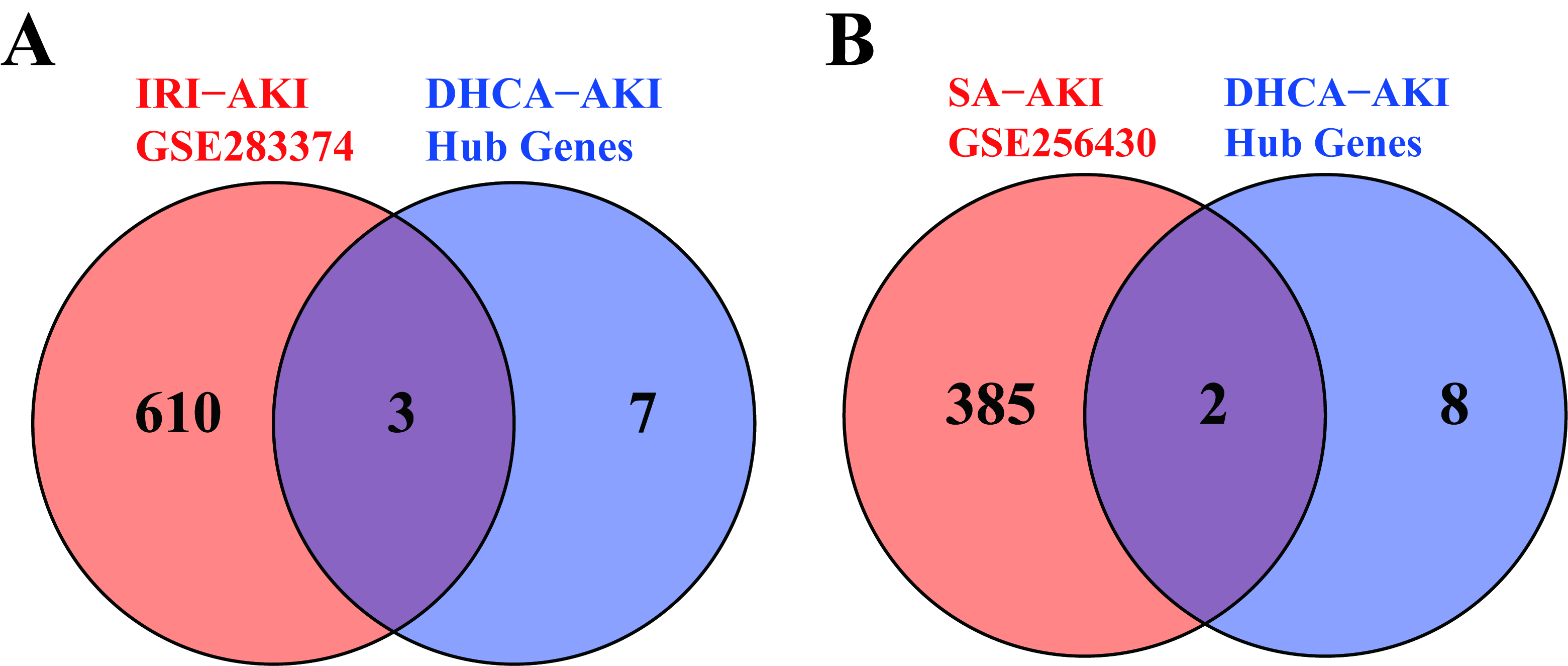


**Supplementary Figure S3** Comparison of DEGs in DHCA-AKI with those in other types of AKI. (A) Comparison of DEGs in DHCA-AKI with IRI induced AKI. (B) Comparison of DEGs in DHCA-AKI with SA-AKI. DEGs: differentially expressed genes; DHCA: deep hypothermic circulatory arrest; AKI: acute kidney injury; SA-AKI: sepsis-associated AKI.

# Supplementary Table S1 The primer sequences for the ten rat hub genes

|  | Forward primer sequence | Reverse primer sequence |
| --- | --- | --- |
| JUN | CGCACGCTCCTAAACAAACTTTG | GTCGTTTCCATCTTTGCAGTCAT |
| ATF3 | AAGAGCTGAGATTCGCCATCC | ACTTGGCAGCAGCAATTTTGT |
| CEBPB | CGGAACTTGTTCAAGCAGCTG | GGATTGCATCAAGTCCCGAAAC |
| IL-1β | CAACTGTCCCTGAACTCAACTGT | GAGATGCTGCTGTGAGATTTGAA |
| NFκBIA | CGGAGTCAGAATTCACGGAAGAT | ACAACAGCTGAATCACCACAATAG |
| PTGS2 | ACAACAACTCCATCCTCCTTGAA | TCATCTCTCTGCTCTGGTCAATG |
| CXCL1 | CTGCACCCAAACCGAAGTCATAG | CCATCGGTGCAATCTATCTTCTTTC |
| NFκBIZ | CTGCTGCACATCCGAAGTAATAA | GTGGAGATGTTTGTGGTGATGTC |
| ZFP36 | TCTGCCATCTACGAGAGCCTTAT | GGAGTCCGATGAGTTTATGTTCCA |
| TNFAIP3 | GAATTTGTGGAGACGGGACTTTG | TGAGGATGTTGCTGAGGACAAATA |

**Supplementary Table S2** Antibodies used in Immunohistochemistry, Western blot, and Immunofluorescence analyses.

| Target Protein | primary antibodies (Supplier, Catalog Number) |
| --- | --- |
| JUN | C-jun Rabbit pAb (Bioss, bs-0670R) |
| ATF3 | [ATF3 Rabbit pAb](http://www.biosschina.com/productDetail?goods_id=20142) (Bioss, bs-0519R) |
| CEBPB | CEBP Beta Rabbit pAb (Bioss, bs-1396R) |
| IL-1β | IL-1 Beta Rabbit pAb (Bioss, bs-0812R) |
| NFκBIA | IKB alpha Rabbit pAb (Bioss, bs-1287R) |
| PTGS2 | PTGS2 Rabbit pAb (Bioss, bs-0732R) |
| CXCL1 | CXCL1 Rabbit pAb (Bioss, bs-10234R) |
| NFκBIZ | ActivAb^TM^ Anti-NFKBIZ Polyclonal Antibody (Solarbio, K002023P) |
| ZFP36 | ZFP36 Rabbit pAb (Bioss, bs-20893R) |
| TNFAIP3 | ActivAb^TM^ Anti-TNFAIP3 Polyclonal Antibody (Solarbio, K003240P) |

# Supplementary Table S3 The primer sequences for the ten human hub genes

|  | Forward primer sequence | Reverse primer sequence |
| --- | --- | --- |
| JUN | CCTTGAAAGCTCAGAACTCGGAG | TGCTGCGTTAGCATGAGTTGGC |
| ATF3 | CGCTGGAATCAGTCACTGTCAG | CTTGTTTCGGCACTTTGCAGCTG |
| CEBPB | AGAAGACCGTGGACAAGCACAG | CTCCAGGACCTTGTGCTGCGT |
| IL-1β | CCACAGACCTTCCAGGAGAATG | GTGCAGTTCAGTGATCGTACAGG |
| NFκBIA | TCCACTCCATCCTGAAGGCTAC | CAAGGACACCAAAAGCTCCACG |
| PTGS2 | CGGTGAAACTCTGGCTAGACAG | GCAAACCGTAGATGCTCAGGGA |
| CXCL1 | AGCTTGCCTCAATCCTGCATCC | TCCTTCAGGAACAGCCACCAGT |
| NFκBIZ | CCGATTCGTTGTCTGATGGACC | GCACTGCTCTCCTGTTTGGGTT |
| ZFP36 | GCTATGTCGGACCTTCTCAGAG | CCTGGAGGTAGAACTTGTGACAG |
| TNFAIP3 | CTCAACTGGTGTCGAGAAGTCC | TTCCTTGAGCGTGCTGAACAGC |

**R scripts for the WGCNA analysis**

dir.create("wgcna",recursive = T)

#引用包

library(limma)

library(WGCNA)

load("Data.Rdata")

#读取输入文件，并对输入文件整理

data.n=express_tpm[,group_list == "normal"]

data.t=express_tpm[,group_list == "DHCA"]

data=cbind(data.n,data.t)

data=data[apply(data,1,sd)>0.5,] #去除波动小的基因

#提取样品的分组信息(对照组和实验组)

Type=group_list

conCount=length(Type[Type=="normal"])

treatCount=length(Type[Type=="DHCA"])

datExpr0=t(data)

###检查缺失值

gsg = goodSamplesGenes(datExpr0, verbose = 3)

if (!gsg$allOK){

# Optionally, print the gene and sample names that were removed:

if (sum(!gsg$goodGenes)>0)

printFlush(paste("Removing genes:", paste(names(datExpr0)[!gsg$goodGenes], collapse = ", ")))

if (sum(!gsg$goodSamples)>0)

printFlush(paste("Removing samples:", paste(rownames(datExpr0)[!gsg$goodSamples], collapse = ", ")))

# Remove the offending genes and samples from the data:

datExpr0 = datExpr0[gsg$goodSamples, gsg$goodGenes]

}

###样品聚类

sampleTree = hclust(dist(datExpr0), method = "average")

pdf(file = "wgcna/1_sample_cluster.pdf", width = 8, height = 8)

par(cex = 0.6)

par(mar = c(0,4,2,0))

plot(sampleTree, main = "Sample clustering to detect outliers", sub="", xlab="", cex.lab = 1.5, cex.axis = 1.5, cex.main = 2)

###剪切线

abline(h = 33, col = "red")

dev.off()

###删除剪切线以下的样品

# clust = cutreeStatic(sampleTree, cutHeight=35, minSize=33)

# table(clust)

# keepSamples = (clust==1)

# datExpr0 = datExpr0[keepSamples, ]

###准备样品的临床数据

traitData=data.frame(Control=c(rep(1,conCount),rep(0,treatCount)),

Treat=c(rep(0,conCount),rep(1,treatCount)))

row.names(traitData)=colnames(data)

fpkmSamples = rownames(datExpr0)

traitSamples =rownames(traitData)

sameSample=intersect(fpkmSamples,traitSamples)

datExpr0=datExpr0[sameSample,]

datTraits=traitData[sameSample,]

###再次对样品进行聚类,得到样品聚类的热图

sampleTree2 = hclust(dist(datExpr0), method = "average")

traitColors = numbers2colors(datTraits, signed = FALSE)

pdf(file="wgcna/2_sample_heatmap.pdf", width=9, height=7)

plotDendroAndColors(sampleTree2, traitColors,

groupLabels = names(datTraits),

main = "Sample dendrogram and trait heatmap")

dev.off()

###power值的散点图

enableWGCNAThreads() #多线程工作

powers = c(1:20) #幂指数范围1:20

sft = pickSoftThreshold(datExpr0, powerVector = powers, verbose = 5)

pdf(file="wgcna/3_scale_independence.pdf", width=9, height=5)

par(mfrow = c(1,2))

cex1 = 0.9

###拟合指数与power值散点图

plot(sft$fitIndices[,1], -sign(sft$fitIndices[,3])*sft$fitIndices[,2],

xlab="Soft Threshold (power)",ylab="Scale Free Topology Model Fit,signed R^2",type="n",

main = paste("Scale independence"));

text(sft$fitIndices[,1], -sign(sft$fitIndices[,3])*sft$fitIndices[,2],

labels=powers,cex=cex1,col="red");

abline(h=0.8, col="red") #可以修改

###平均连通性与power值散点图

plot(sft$fitIndices[,1], sft$fitIndices[,5],

xlab="Soft Threshold (power)",ylab="Mean Connectivity", type="n",

main = paste("Mean connectivity"))

text(sft$fitIndices[,1], sft$fitIndices[,5], labels=powers, cex=cex1,col="red")

dev.off()

###邻接矩阵转换

sft #查看最佳power值

softPower = 7

adjacency = adjacency(datExpr0, power = softPower)

softPower

###TOM距离矩阵

TOM = TOMsimilarity(adjacency)

dissTOM = 1-TOM

###基因聚类

geneTree = hclust(as.dist(dissTOM), method = "average");

pdf(file = "wgcna/4_gene_clustering.pdf", width=8, height=6)

plot(geneTree, xlab="", sub="", main = "Gene clustering on TOM-based dissimilarity",

labels = FALSE, hang = 0.04)

dev.off()

###动态的模块识别

minModuleSize = 60 #模块基因数目(每个模块至少包含60个基因)

dynamicMods = cutreeDynamic(dendro = geneTree, distM = dissTOM,

deepSplit = 2, pamRespectsDendro = FALSE,

minClusterSize = minModuleSize);

table(dynamicMods)

dynamicColors = labels2colors(dynamicMods)

table(dynamicColors)

pdf(file = "wgcna/5_Dynamic_Tree.pdf", width=8, height=6)

plotDendroAndColors(geneTree, dynamicColors, "Dynamic Tree Cut",

dendroLabels = FALSE, hang = 0.03,

addGuide = TRUE, guideHang = 0.05,

main = "Gene dendrogram and module colors")

dev.off()

###对模块进行聚类,找出相似模块聚类

MEList = moduleEigengenes(datExpr0, colors = dynamicColors)

MEs = MEList$eigengenes

MEDiss = 1-cor(MEs);

METree = hclust(as.dist(MEDiss), method = "average")

pdf(file = "wgcna/6_Clustering_module.pdf", width=7, height=6)

plot(METree, main = "Clustering of module eigengenes",

xlab = "", sub = "")

MEDissThres = 0.08 #>>>>>>>>>>>剪切高度可修改

abline(h=MEDissThres, col = "red")

dev.off()

###相似模块合并

merge = mergeCloseModules(datExpr0, dynamicColors, cutHeight = MEDissThres, verbose = 3)

mergedColors = merge$colors

mergedMEs = merge$newMEs

pdf(file = "wgcna/7_merged_dynamic.pdf", width=8, height=6)

plotDendroAndColors(geneTree, mergedColors, "Merged dynamic",

dendroLabels = FALSE, hang = 0.03,

addGuide = TRUE, guideHang = 0.05,

main = "Gene dendrogram and module colors")

dev.off()

moduleColors = mergedColors

table(moduleColors)

colorOrder = c("grey", standardColors(50))

moduleLabels = match(moduleColors, colorOrder)-1

MEs = mergedMEs

###绘制模块与临床性状相关性的热图

nGenes = ncol(datExpr0)

nSamples = nrow(datExpr0)

moduleTraitCor = cor(MEs, datTraits, use = "p")

moduleTraitPvalue = corPvalueStudent(moduleTraitCor, nSamples)

pdf(file = "wgcna/8_Module_trait.pdf", width=5.5, height=5.5)

textMatrix = paste(signif(moduleTraitCor, 2), "\n(",

signif(moduleTraitPvalue, 1), ")", sep = "")

dim(textMatrix) = dim(moduleTraitCor)

par(mar = c(5, 10, 3, 3))

labeledHeatmap(Matrix = moduleTraitCor,

xLabels = names(datTraits), #X轴的标签

yLabels = names(MEs), #Y轴的标签

ySymbols = names(MEs),

colorLabels = FALSE,

colors = blueWhiteRed(50), #图形的颜色

textMatrix = textMatrix, #图形展示的文本信息

setStdMargins = FALSE,

cex.text = 0.75, #字体的大小

zlim = c(-1,1), #相关系数的范围

main = paste("Module-trait relationships")) #图形的标题

dev.off()

###计算MM和GS值

modNames = substring(names(MEs), 3)

geneModuleMembership = as.data.frame(cor(datExpr0, MEs, use = "p"))

MMPvalue = as.data.frame(corPvalueStudent(as.matrix(geneModuleMembership), nSamples))

names(geneModuleMembership) = paste("MM", modNames, sep="")

names(MMPvalue) = paste("p.MM", modNames, sep="")

traitNames=names(datTraits)

geneTraitSignificance = as.data.frame(cor(datExpr0, datTraits, use = "p"))

GSPvalue = as.data.frame(corPvalueStudent(as.matrix(geneTraitSignificance), nSamples))

names(geneTraitSignificance) = paste("GS.", traitNames, sep="")

names(GSPvalue) = paste("p.GS.", traitNames, sep="")

###输出模块重要性的图形

y=datTraits[,1]

GS1=as.numeric(cor(y, datExpr0, use="p"))

GeneSignificance=abs(GS1)

ModuleSignificance=tapply(GeneSignificance, mergedColors, mean, na.rm=T)

pdf(file = "wgcna/9_GeneSignificance.pdf", width=11, height=7)

plotModuleSignificance(GeneSignificance, mergedColors)

dev.off()

###对模块进行循环，得到每个模块的散点图

trait="Treat"

traitColumn=match(trait,traitNames)

for (module in modNames){

column = match(module, modNames)

moduleGenes = moduleColors==module

if (nrow(geneModuleMembership[moduleGenes,]) > 1){

outPdf=paste("wgcna/10_", trait, "_", module,".pdf",sep="")

pdf(file=outPdf, width=7, height=7)

par(mfrow = c(1,1))

verboseScatterplot(abs(geneModuleMembership[moduleGenes, column]),

abs(geneTraitSignificance[moduleGenes, traitColumn]),

xlab = paste("Module Membership in", module, "module"),

ylab = paste("Gene significance for ",trait),

main = paste("Module membership vs. gene significance\n"),

cex.main = 1.2, cex.lab = 1.2, cex.axis = 1.2, col = module)

abline(v=0.8, h=0.5, col="red")

dev.off()

}

}

###输出GS_MM数据

probes = colnames(datExpr0)

geneInfo0 = data.frame(probes= probes,

moduleColor = moduleColors)

for (Tra in 1:ncol(geneTraitSignificance)){

oldNames = names(geneInfo0)

geneInfo0 = data.frame(geneInfo0, geneTraitSignificance[,Tra],

GSPvalue[, Tra])

names(geneInfo0) = c(oldNames,names(geneTraitSignificance)[Tra],

names(GSPvalue)[Tra])

}

for (mod in 1:ncol(geneModuleMembership)){

oldNames = names(geneInfo0)

geneInfo0 = data.frame(geneInfo0, geneModuleMembership[,mod],

MMPvalue[, mod])

names(geneInfo0) = c(oldNames,names(geneModuleMembership)[mod],

names(MMPvalue)[mod])

}

geneOrder =order(geneInfo0$moduleColor)

geneInfo = geneInfo0[geneOrder, ]

write.table(geneInfo, file = "wgcna/GS_MM.xls",sep="\t",row.names=F)

###输出每个模块的基因

for (mod in 1:nrow(table(moduleColors))){

modules = names(table(moduleColors))[mod]

probes = colnames(datExpr0)

inModule = (moduleColors == modules)

modGenes = probes[inModule]

write.table(modGenes, file =paste0("wgcna/module_",modules,".txt"),sep="\t",row.names=F,col.names=F,quote=F)

}

###输出每个模块的核心基因

geneSigFilter=0.5 #基因重要性的过滤条件

moduleSigFilter=0.8 #基因与模块相关性的过滤条件

cli="GS.Treat"

for(mol in unique(geneInfo$moduleColor)){

geneInfoMol=geneInfo[geneInfo$moduleColor==mol,]

mmi=paste0("MM", mol)

geneInfoMol2=geneInfoMol[((abs(geneInfoMol[,mmi])>moduleSigFilter) & (abs(geneInfoMol[,cli])>geneSigFilter)),]

write.table(geneInfoMol2[,1], file =paste0("wgcna/hubGenes_",mmi,".txt"),sep="\t",row.names=F,col.names=F,quote=F)

}
